# Significant Improvement in Rat Kidney Cold Storage Using UW Organ Preservation Solution Supplemented With the Immediate-Acting PrC-210 Free Radical Scavenger

**DOI:** 10.1097/TXD.0000000000001032

**Published:** 2020-07-15

**Authors:** Bret M. Verhoven, Aos S. Karim, Natalie M. Bath, Carol J. Sarabia Fahl, Nancy A. Wilson, Robert R. Redfield, William E. Fahl

**Affiliations:** 1 Division of Organ Transplant, Department of Surgery, University of Wisconsin-Madison, Madison, WI.; 2 Wisconsin Institutes for Medical Research, Department of Oncology, University of Wisconsin-Madison, Madison, WI.

## Abstract

Ischemia-reperfusion injury, including injury from warm- and cold-ischemia (CI) organ storage, remains a significant problem for all solid organ transplants. Suppressing CI damage would reduce delayed graft function and increase the donor organ pool size. PrC-210 has demonstrated superior prevention of damage in several preclinical studies as an immediate-acting free-radical scavenger. Here, we describe its profound efficacy in suppressing CI injury in a rat kidney model.

**Methods.:**

Kidneys in 300 gm Sprague-Dawley rats were perfused in situ with UW solution with or without added PrC-210 and then stored at 4°C in the same solution for 0 to 48 hours. When procured, kidney-activated caspase-3 level (a marker of cell death) was measured, and direct histological analysis of kidneys was performed to assess PrC-210 protective efficacy. In vitro analyses of PrC-210-conferred protection to isolated rat kidneys or naked DNA were also performed.

**Results.:**

A single 15 seconds in situ perfusion of kidneys with 20 mmol/L PrC-210 in UW solution resulted in significant reductions in (1) 30-hour CI–induced kidney-activated caspase level (*P* < 0.0001); activated caspase was reduced to levels not significantly different than control activated caspase levels seen in unperturbed kidneys, (2) 30-hour CI–induced renal Tubular Injury Scores (*P* = 0.0004) where brush border and tubular necrosis were markedly reduced, (3) PrC-210 conferred 100% protection against ·OH damage to naked DNA and isolated kidney mitochondria while current UW solution antioxidants were without protective effect.

**Conclusions.:**

A single PrC-210-UW solution perfusion of rat kidneys upon removal from the rat profoundly reduced caspase and renal tubular injury in kidneys exposed to 30 hours of CI organ storage. These findings support further development of the PrC-210 molecule to suppress or to prevent ischemia-reperfusion injury in organ transplant and other ischemia-reperfusion injury settings.

End-stage renal failure causes >1.2 million deaths annually in the world.^1^ Kidney transplantation is the preferred treatment for patients with end-stage renal disease. Over 70 000 kidney transplants are performed each year in the world. The development of UW solution (Viaspan) and its commercialization in 1986 transformed the organ transplantation field. UW solution significantly extends organ preservation time thereby increasing the donor pool.^2^

Despite UW solution, ischemia-reperfusion (IR) injury remains a significant problem for kidney transplantation; IR-injury manifests primarily as delayed graft function. About one-third of all kidney transplants will develop delayed graft function (DGF), which is defined as the need for dialysis within 1 week of kidney transplantation; this failure rate increases to as high as 50% in kidneys donated after circulatory death.^3–5^ DGF is a well-established risk factor for inferior graft survival.^6,7^ DGF also leads to increased resource utilization and expense in the immediate posttransplant setting as one awaits the return of kidney function.^8^ Thus, an important, unmet need in kidney transplantation, actually all organ transplantation, is the prevention of IR injury. As IR-injury implies, organ damage occurs during both (1) the warm and then cold-ischemia (CI)^9^/hypoxia of prolonged cold organ storage and (2) the warm-reperfusion immediately following organ implant.

Although cold storage of kidneys in UW solution greatly extends transit times, several studies have described the risk of DGF associated with extended CI time.^5,10,11^ Ojo et al^12^ reported a 23% increase in DGF for every 6 hours increase in CI time. Others show a substantial increase in DGF with CI time at or >30 hours.^13^

Nydam et al^9^ report that the mechanism by which extended CI results in DGF is not known. However, Poyton et al^14^ describe that as follows:

1.Under hypoxic conditions, which include kidney CI, the kidney mitochondrial respiratory chain produces both nitric oxide (NO·)^15^ and reactive oxygen species (ROS), which both result in toxic chemical modification of cellular nucleic acids, proteins, and lipids.^16^
2.Under oxidative conditions, which include kidney warm-reperfusion, the kidney mitochondrial respiratory chain, as well as other post-ischemia cell mechanisms, produce a bolus of ROS. This warm-reperfusion-associated ROS overproduction results in the oxidation of cellular nucleic acids, proteins, lipids, glutathione, and more.^17,18^ This process is commonly referred to as oxidative stress.

PrC-210 is the prototype of a new family of direct-acting, small molecule aminothiol ROS scavengers,^19–21^ which can be administered orally, IV, or topically, and it has no measurable nausea/emesis nor hypotension side effects.^21^ PrC-210 is not an antioxidant. Unlike traditional antioxidants that act indirectly over hours to days via NrF-2 to activate expression of protective genes,^22^ PrC-210 directly scavenges ROS to confer 100% protection in seconds to minutes.^23,24^ PrC-210 is the most effective, direct-acting, ROS scavenger in existence today.^23,25^ To determine if PrC-210 can suppress the kidney cell death that occurs during the extended CI required in human kidney transplant, we measured the ability of PrC-210, administered in situ as a single perfusion of a rat donor kidney and also present during the 30 hour 4°C cold storage, to reduce the degree of kidney caspase and kidney tissue destruction over a 30-hour period of kidney CI storage.

## MATERIALS AND METHODS

### Animals

Three hundred gram Sprague-Dawley outbred rats were purchased from Envigo (Indianapolis, IN) and housed in the University of Wisconsin Laboratory Animal Facility. All procedures were performed in accordance with the Animal Care and Use Policies at the University of Wisconsin. Animal health including animal deaths, room temperature, 12-hour light/dark cycles, and cage cleaning among other sanitation duties were performed daily by animal care staff. Food and water were available ad libitum. This research was prospectively approved by School of Medicine and Public Health Institutional Animal Care and Use Committee at the University of Wisconsin (Protocol no. B00000588). All groups contained 4 to 6 animals.

### Materials

Synthesis of the PrC-210 HCl aminothiol is described separately.^26,27^ PrC-210 HCl crystals are stored under a nitrogen atmosphere at −20°C, and even with routine thawing, use, and restorage, crystalline PrC-210 is completely stable for >4 years by mass spectrometry analysis. Other chemical reagents were obtained from Sigma Aldrich (St. Louis, MO).

### Experimental and Surgical Procedure

After double ligation of the aorta and surgical section of both renal veins, both rat kidneys were perfused simultaneously in situ using 5 mL of room temperature UW solution (over a 15-s period). The perfusate was either UW solution alone (for 0 h and 0 mmol/L groups), or UW solution to which crystalline PrC-210 (to achieve 0 to 40 mmol/L) had been added, dissolved immediately, and then pH-adjusted to the starting UW solution pH of 7.4 by adding 0.0619 µL 5N NaOH per µmol of PrC-210 HCL salt (FW: 220). Following in situ perfusion, kidneys were surgically removed, dropped into 5 mL of UW solution containing the same PrC-210 concentration as the perfusate, and then stored on ice at 4°C for 0 to 48 hours before retrieval.

### Renal Assessments

Activated caspase 3 and 7 activity in kidney homogenate supernates was determined using the Apo-ONE fluorescent substrate (Promega, Madison, WI).^24^ Briefly, thawed kidneys were mixed with an 8-fold excess of lysis buffer containing 50 mmol/L Na HEPES, pH 7.4, 100 mmol/L NaCl, 1 mmol/L ethylene diamine tetra-acetic acid, 10 mmol/L dithiothreitol, 10% glycerol and homogenized at 4°C for 30 seconds with an Omni tissue homogenizer. The kidney homogenate was centrifuged at 4°C (16 000*g*) in an Eppendorf microfuge for 20 minutes. The supernates were immediately assayed for caspase activity, and protein content by the Bradford method using BSA as the standard. The activated caspase assay was performed as follows: 5 µL supernate (≈40 μg of supernate protein) was diluted to a total volume of 50 µL with the above lysis buffer, was mixed with 50 μL of the undiluted Apo-ONE substrate in the well of a black, opaque, 96 well plate to initiate the 60-minute reaction. Plates were shaken at 200 RPM at 37°C for 60 minutes. The DEVD (Asp-Glu-Val-Asp peptide) caspase substrate peptide cleavage was measured using a BMG Clariostar fluorescent plate reader at an excitation wavelength of 499 nm and an emission wavelength of 521 nm. A caspase standard was included in each experiment.

### Semiquantification of Kidney Histology

Half-kidneys were fixed in 10% formalin and embedded in paraffin; sections were then mounted and stained with hematoxylin-eosin. Slides were scanned using a 20X objective in an Aperio Digital Pathology Slide Scanner. Slides were assigned a blinded number, and 5, random, nonoverlapping digital images of renal tubules were taken at the interface between the medulla and the cortex from each H/E slide spanning the entire kidney length. Care was taken to not include large vessel lumens and glomeruli. Automated quantification in each 10X kidney image was performed using a custom macro written in ImageJ software (https://imagej.nih.gov/ij/index.html). Briefly, each 10X kidney tubule image was separated into red, blue, and green channels using the Colour deconvolution plugin by Ruifrok et al (https://imagej.net/Colour_Deconvolution) using the H&E algorithm. The image threshold was obtained using Otsu’s algorithm. The red channel was used to quantify tubular thickness including brush border; healthy tubules had a robust cytoplasmic staining with abundant nuclei, while injured tubules or necrotic tubules had decreased cytoplasmic staining due to rupture and loss of nuclei. Nuclei were quantified in the blue channel after running the despeckle, watershed, and analyze particles (10 pixels to infinity; circularity 0.25–1.0) algorithms. The ratio of blue nuclear pixels to red tubules was determined to yield a Rat Kidney Tubular Necrosis Score. Scores were then averaged and plotted using Graphpad Prism.

### PrC-210 Versus Other Molecule Protection of Naked Plasmid DNA

In a previous report^25^ 6 mmol/L PrC-210 was shown to confer complete protection of naked plasmid DNA against a bolus hydroxyl radical insult. To compare PrC-210 ROS-scavenging protective efficacy against the other antioxidants and protective molecules, which either (1) have been integral components of UW solution since its origin in 1985^2^ (ie, glutathione, adenosine, and allopurinol) or (2) are included in the new BUPS Preservation Solution^28^ (ie, taurine, N-acetylcysteine, and ascorbic acid), we performed a head-to-head comparison of each molecule when added at 6 mmol/L to pGEM plasmid DNA (750 ng) 10 minutes before administering a bolus ·OH insult (ie, 90 sec irradiation in an X-RAD 320 irradiator). Immediately following the 90 seconds ·OH pulse, triplicate samples of the irradiated plasmid DNA (200 ng each) were electrophoresed on a 1% agarose gel in Tris-acetate buffer for 90 minutes at 60 volts. Gels were stained with ethidium bromide, digitally imaged, and supercoiled versus nicked/·OH damaged DNA band intensities were quantified using BioRad ImageLab software. In a previous report,^24^ the ·OH insult was administered by combining H2O2 with 60 seconds UV-irradiation. Because several of the test molecules strongly absorb UV light, we could not use UV-irradiation here.

### Rat Kidney Mitochondria

The purified mitochondrial fraction was prepared from homogenized rat kidneys by a standard centrifugation technique.^29^ The purified mitochondria were suspended in 0.15 mol/L Tris HCl buffer, pH 7.4.

To determine whether addition of exogenous PrC-210 suppresses formation of ROS-oxidized fatty acids in mitochondria, in a 76 µL reaction volume (in a 0.5 mL Eppendorf tube), we added: 44 µL purified mitochondria, 10 µL 0.15 mol/L Tris (pH 7.4), 10 µL PrC-210 dilution or water (PrC-210 was added 10 min before the Fe^++^ + ADP + H_2_O_2_ ·OH generator), 6 µL containing FeCl2 (6 mmol/L; FW:127) and adenosine 5′-diphosphate sodium salt (24 mmol/L; FW: 427), and 6 µL 0.036% H_2_O_2_.^30^ After 10 minutes at 37°C, 125 µL 20% trichloroacetic acid and 125 µL thiobarbituric acid (0.67% in water) were added to each 0.5 mL Eppendorf tube and vortexed for 5 seconds. Tubes were then incubated at 90°C for 40 minutes, centrifuged at full speed in a microfuge for 4 minutes, and 250 µL of supernatant from each tube was transferred to a well of a clear 96 well plate. Absorbance at 532 nm was read in each well. The malondialdehyde (MDA) standard curve was constructed by adding ethanol dilutions of 1,1,3,3-tetramethoxypropane (Sigma Aldrich) to tubes containing the same trichloroacetic acid + thiobarbituric acid volumes as above to achieve final 1,1,3,3-tetramethoxypropane concentrations of 0.33 to 9.8 µmol/L.

To determine whether the addition of exogenous PrC-210 suppresses ROS-induced fragmentation of mitochondrial DNA,^29^ in a 25 µL reaction volume (in a polymerase chain reaction tube), we added: 10 µL purified mitochondria, 5 µL PrC-210 dilution or water (PrC-210 was added 10 min before the Fe^++^ + ADP ·OH generator), and 10 µL containing FeCl_2_ (2.5 mmol/L; FW:127) and adenosine 5′-diphosphate sodium salt (10 mmol/L; FW: 427). After 20 minutes at 37°C, 10 µL of the reaction was mixed with 5 µL of 6 X gel-loading dye containing 0.3% sodium dodecyl sulfate (SDS); tubes sat in 60°C water for 1 minutes, 10 µL was then loaded into a well of a 1% agarose TAE gel, and after 60 minutes at 60 volts, gels were stained and photographed. A minimum of 3 replicates were done for each assay point to enable statistical comparison.

### Statistics

Data are expressed as means ± STDs. One-tailed Student *t* tests were used to determine statistical difference and *P* values using GraphPad Prism 7.03 software. *P* values <0.05 were considered significant.

## Results

### Time-Dependent Kidney Cell Death During Storage in Cold UW Solution

**FIGURE 1.**
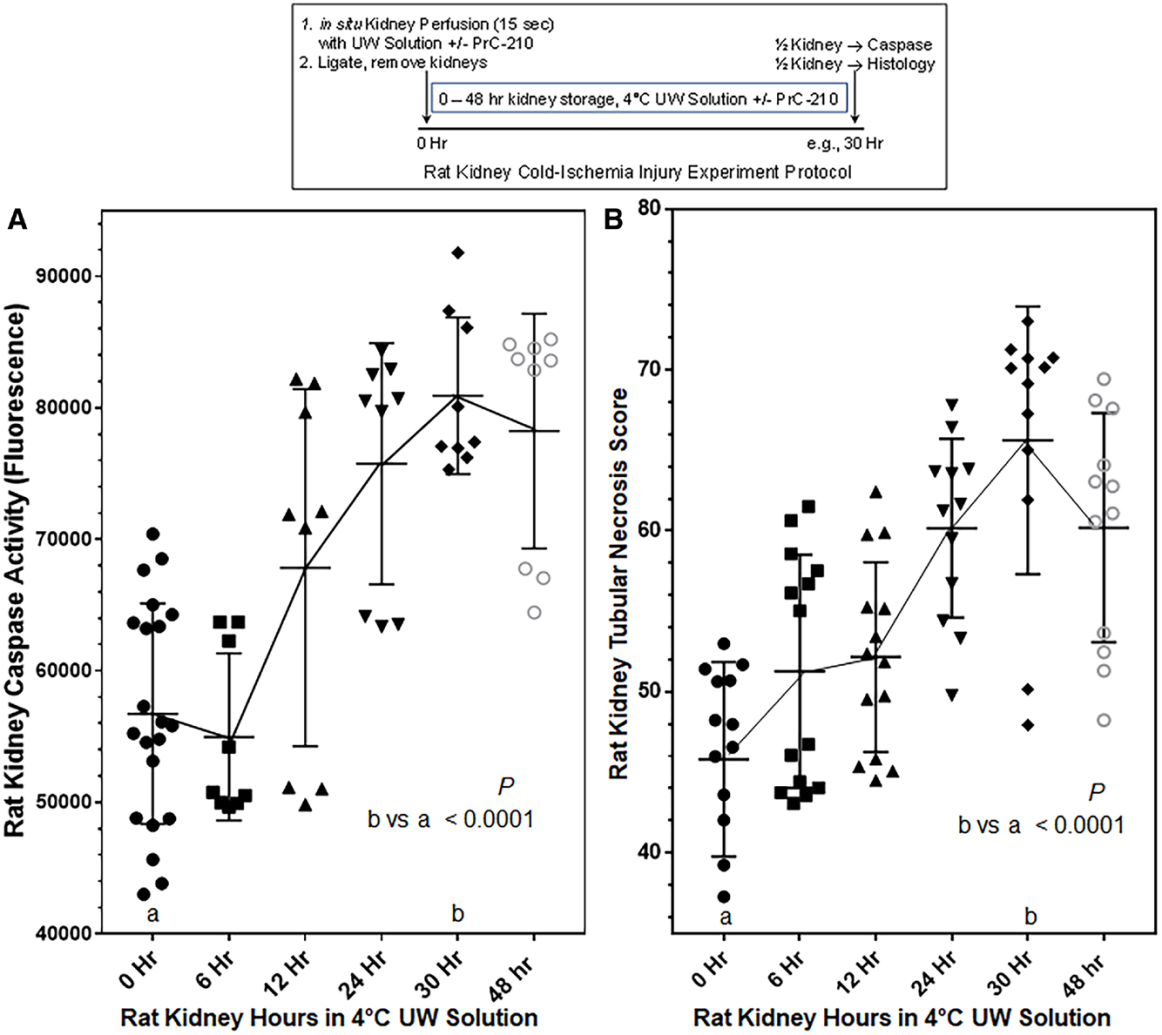
A, Time-dependent cell death in rat kidneys flushed and then stored in 4°C UW solution for 0–48 h. Kidneys were perfused in situ in rats with room temperature UW solution, the blood vessels ligated, and the kidneys were then stored in 4°C UW solution for the indicated times. Kidney supernatant activated caspase activity was measured as described in Materials and Methods in a 60 min reaction. B, Rat Kidney Tubular Necrosis Scores (see Figure 2 and Materials and Methods for scoring procedure) increased with kidney storage time in 4°C UW solution. A minimum of 6 kidneys were studied at each time point; individual assay values are plotted with STDs to show sample variability.

To determine the extent and locations of kidney cell death during CI storage, following aortic ligations and severing the renal veins, we measured activated caspase activity and quantified injury in histology. Activated caspase activity in half-kidney homogenization supernatants increased after 30-hour storage by 51% over the 0-time kidney control activated caspase background (Figure 1A) that is associated with normal kidney cell differentiation and death. Kidney-activated caspase activity was measured as a surrogate marker of CI-induced cell death.

**FIGURE 2.**
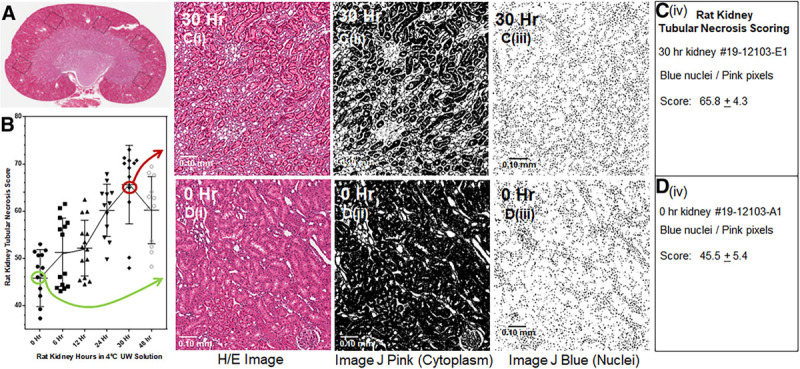
Kidney histology following storage in 4°C UW solution. Blinded 20X scanned images of each kidney were analyzed. Briefly, (A) operator first randomly collected 5 sample images of renal tubules (A, boxes) from each kidney. Using an ImageJ macro (written by A.K.), H/E images (eg, C (i) and D (i)) were analyzed, and above-threshold pink pixels were selected and enumerated (eg, C (ii) and D (ii)), and above-threshold blue pixels were selected and enumerated (eg, C (iii) and D (iii)). Rat Kidney Tubular Necrosis Scores (C (iv) and D (iv)) were assigned as shown.

Renal tubule histology was acquired and scored for each cold storage kidney (Figure 1B summarizes scores). Figure 2A–D show kidney histology. Figure 2D (i) shows normal renal tubule structure at zero time, and Figure 2C (i) shows that after 30-hour cold storage in UW solution there is marked tubular necrosis, with clear loss of tubule and brush border structure but retention of most of the hematoxylin-stained tubule cell nuclei, albeit they are larger and have a speckled appearance indicating chromatin condensation. Figure 2C (ii) and D (ii) show Image J semiquantification in which total pink (eosin-stained) pixels or in Figures 2C (iii) or D (iii) total blue (hematoxylin-stained) pixels have been quantified. This pixel quantification was performed on 5 randomly selected, nonoverlapping tubule images (eg, Figure 2A). The time-dependent increase in tubular necrosis score (Figure 1B) clearly reflected the same CI time-dependent increases in cell death as measured by caspase activity (Figure 1A).

### PrC-210 Protection of Naked DNA

**FIGURE 3.**
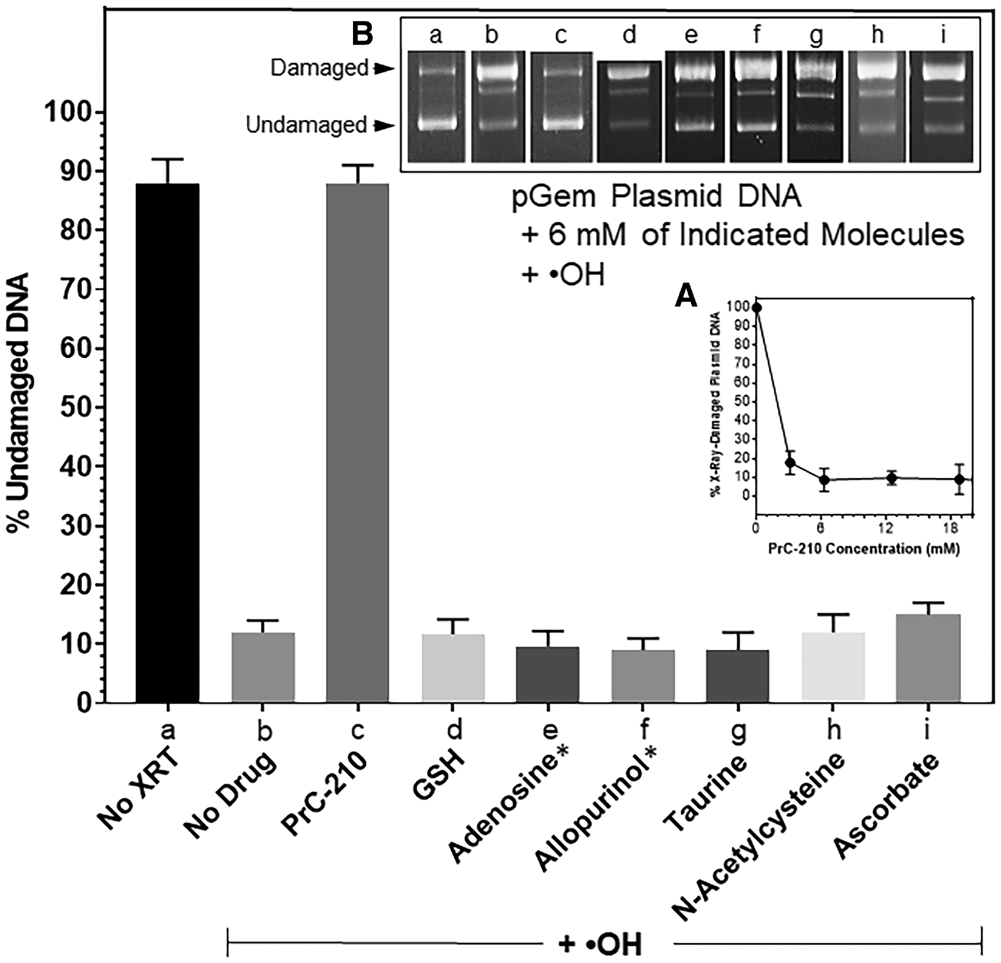
Agarose gel separation of supercoiled (undamaged) and nicked/·OH-damaged (damaged) forms of pGEM plasmid DNA after exposure of the plasmid DNA to a 90 s pulse of ·OH generated x-irradiation (XRT). Purified, pGEM DNA (86% supercoiled) was incubated with buffer alone (lane a) or 6 mmol/L of each of the indicated protective molecules (lanes b–i) for 10 min before the 90 s x-irradiation. Aliquots of each reaction were then immediately electrophoresed, stained with ethidium bromide (EtBr), and digitally imaged. Three replicate reactions and gels were done. Inset A: Previous studies^aa^ showed that 6 mmol/L PrC-210 conferred complete protection of naked plasmid DNA against ·OH so all molecules were compared at 6 mmol/L. Inset B: Individual EtBr-stained lanes from agarose gels. *6 mmol/L adenosine and allopurinol solutions were titrated to pH 11 with NaOH to solubilize the drug material before adding to buffer + pGEM DNA.

The gel-based assay of ·OH-induced plasmid DNA breaks, in which a 90-second pulse of ·OH was generated by *x*-ray, demonstrated that PrC-210 provided 100% suppression of the ·OH insult that otherwise induced >95% damage to the naked plasmid DNA.^23^ Because several of the comparison molecules absorb UV light, the H_2_O_2_ + UV light ·OH generator used in an earlier report^24^ was replaced here with 90-second x-irradiation to produce the ·OH insult. In a previous titration comparison of antioxidants,^23^ 6 mmol/L PrC-210 was found to confer complete protection (Figure 3A), so the molecules were compared here by addition at 6 mmol/L to DNA 10 minutes before the 90 seconds ·OH insult (Figure 3B). Under these comparison conditions, none of the current UW solution antioxidants (ie, glutathione, adenosine, and allopurinol)^28^ or proposed antioxidants in the new BUPS Solution^28^ (ie, taurine, N-acetylcysteine, and ascorbic acid) showed any protection, while PrC-210 showed 100% protection of the at-risk plasmid DNA.

### PrC-210 Protection of Rat Kidney Mitochondria

**FIGURE 4.**
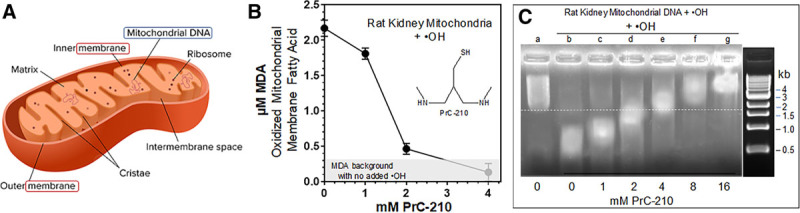
A, Diagram of mitochondrial structure. B, PrC-210 dose-dependent suppression of malondialdehyde (MDA) formation in rat kidney mitochondria treated with the ·OH generator (Fe^++^ + adenosine + H_2_O_2_). PrC-210 was added at indicated concentrations to buffer plus mitochondria 10 min before addition of generator. After 20 min incubation at 37°C, trichloroacetic acid and thiobarbituric acid were added; after 40 min incubation at 90°C, light absorbance at 532 nm was read using 300 µL reaction aliquots (Materials and Methods). C, PrC-210 dose-dependent suppression of mitochondrial DNA fragmentation induced by ·OH generator. PrC-210 was added at indicated concentrations to buffer plus mitochondria 10 min before addition of generator. After 20 min incubation at 37°C, a 10 µL reaction aliquot was mixed with 0.1% sodium dodecyl sulfate (SDS) loading dye, heated to 60°C for 1 min, and then electrophoresed and stained with ethidium bromide.

Kidney mitochondrial function affects both (1) levels of reactive oxygen and nitric oxide species generated during CI and (2) survival of kidney parenchymal cells during CI.^14^ Because of this, we isolated mitochondria from rat kidneys and determined whether PrC-210 at achievable pharmacologic concentrations could protect these organelles (Figure 4A) from an ROS insult. In Figure 4B, mitochondria incubated with an ·OH generator, in this case FeCl_2_ plus ADP plus H_2_O_2_ in a Fenton-like reaction,^30^ sustained significant ROS oxidation of mitochondrial membrane fatty acids (measured as MDA^29^), and addition of PrC-210 minutes before the ROS insult reduced the MDA level to background (at ≈2 mmol/L) in a PrC-210 concentration-dependent manner. As a second measure of mitochondria protection, following ROS insult, an aliquot of the mitochondria were solubilized in SDS-containing gel-loading buffer, and mitochondrial DNA was separated, and thus sized, on an agarose gel (Figure 4C). ROS insult clearly reduced the mean size of the DNA (lane b), and addition of PrC-210 prevented the DNA breakage (lanes c–g) in a PrC-210 concentration-dependent manner. Like MDA suppression, ≈2 mmol/L PrC-210 provided clear DNA protection.

### PrC-210 Reduction of CI-Induced Rat Kidney Cell Death in UW Solution Cold Storage

To test its ability to reduce or eliminate the CI-induced kidney cell death observed in Figure 1, PrC-210 at concentrations of 0 to 30 mmol/L (final) was added to UW solution, and about an hour later the surgically-isolated, in situ kidneys were flushed with 5 mL of the augmented UW solution.

**FIGURE 5.**
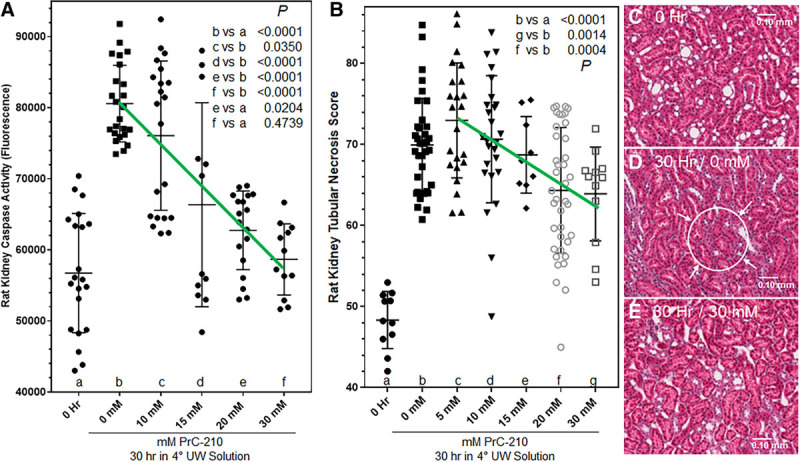
PrC-210 dose-dependent suppression of rat kidney cell death during prolonged (30 h) kidney storage in 4°C UW solution. A, Rat kidneys were flushed once, in situ, over a 30 s period, with room temperature UW solution containing the indicated PrC-210 concentration. Zero-h kidneys were flushed with UW solution. Kidneys were then stored in 4°C UW solution containing the same PrC-210 concentration for 30 h. At 30 h, kidneys were sectioned in half; one-half was frozen in liquid nitrogen before activated caspase assay; one-half was fixed in 10% formalin before histology workup and hematoxylin and eosin staining. Activated caspase assays (A). Histology and assignment of tubular necrosis scores (B–E) were done as described in Materials and Methods. *P* values from Student *t* tests between indicated treatment groups are shown. A minimum of 6 kidneys were studied at each PrC-210 concentration.

Figure 5A shows the substantial increase in kidney-activated caspase at 30 hours in 4°C UW solution alone (lane b versus a). Addition of PrC-210 to UW solution confers a clear concentration-dependent reduction in kidney-activated caspase activity. At ≥20 mmol/L PrC-210, activated caspase is significantly reduced (*P* < 0.0001) from the 30 hour 0 mmol/L PrC-210 control kidneys (lanes c–g versus b), and at 30 mmol/L PrC-210 the activated caspase activity is reduced to the background level seen in the 0-hour control kidneys (lane f versus a; *P* = 0.4739). A representative histology image of a 30-hour kidney with no PrC-210 (Figure 5D) shows profound loss of normal eosin-stained tubule architecture (eg, circle and arrows), and thus a substantial increase in the Tubular Necrosis Score. The histology images (Figures 5B, D, E) clearly support the activated caspase results, and a plot of the Rat Kidney Tubular Necrosis Scores versus PrC-210 concentration in UW solution provides a similar outcome showing a significant PrC-210 concentration-dependent suppression of the 30-hour CI–induced kidney cell death (*P* = 0.0004).

### PrC-210 Reduction of CI-Induced Rat Kidney Cell Death in PBS Cold Storage

**FIGURE 6.**
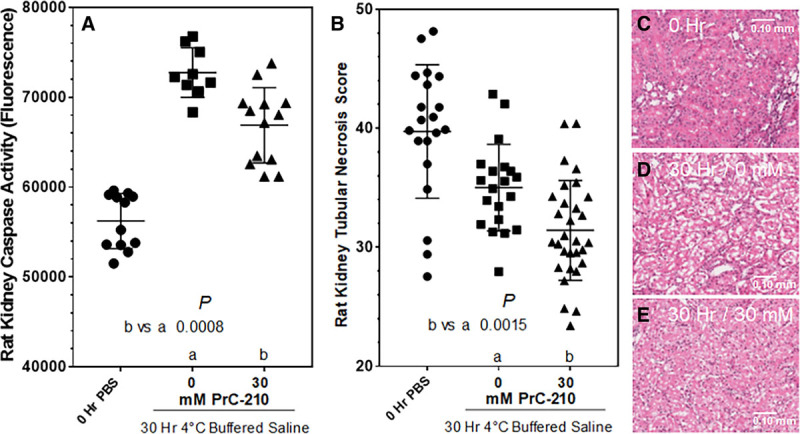
PrC-210 dose-dependent suppression of rat kidney cell death during prolonged (30 h) kidney storage in 4°C Dulbecco PBS (D-PBS). A, Rat kidneys were flushed once, in situ, over a 30 s period, with room temperature D-PBS containing the indicated PrC-210 concentration. Zero-h kidneys were flushed with D-PBS. Kidneys were then stored in 4°C D-PBS containing the same PrC-210 concentration for 30 h. Kidneys were sectioned in half; one-half was frozen in liquid nitrogen before activated caspase assay; one-half was fixed in 10% formalin before histology workup and hematoxylin and eosin staining. Activated caspase assays (A), histology and assignment of tubular necrosis scores (B–E) were done as described in Materials and Methods. *P* values from Student *t* tests between indicated treatment groups are shown. A minimum of 4 kidneys were studied at each PrC-210 concentration.

To determine whether PrC-210 protection was conferred in a preservation solution other than UW solution, PrC-210 crystals were dissolved in Dulbecco PBS (D-PBS) and about an hour later the kidneys were flushed in situ with 5 mL of the augmented D-PBS. Figure 6A shows the substantial increase in kidney-activated caspase at 30 hours in 4°C D-PBS alone, and addition of 30 mmol/L PrC-210 to D-PBS conferred a significant reduction (*P* = 0.0008) in kidney-activated caspase activity. Histology images show normal kidney histology at 0 hour (Figure 6C), profound necrosis and loss of tubule brush border at 30 hours in PBS (Figure 6D), and general retention of tubular architecture with 30 mmol/L PrC-210 present (Figure 6E). The histology images support and replicate the activated caspase results, and the Rat Kidney Tubular Necrosis Scores provide a similar outcome showing a significant PrC-210-conferred suppression of the 30-hour CI–induced kidney cell death (*P* = 0.0015).

## DISCUSSION

IR injury remains a significant problem for kidney and all other solid organ transplants; it manifests primarily as delayed graft function. DGF incidence can be as high as 50% in kidneys donated after circulatory death, and DGF is a well-established risk factor for inferior graft survival. Although cold storage of kidneys in UW solution greatly extends transit times, DGF is clearly associated with extended CI time. We undertook this study to determine if PrC-210 would be effective in preventing the damage induced during the CI that accompanies most organ transplants. Our data demonstrate that (1) kidney cell death from extended CI in UW solution is substantial, (2) a single, 15-second perfusion of PrC-210-containing UW solution upon kidney removal confers a dose-dependent reduction of CI-induced kidney cell death, which when measured by caspase activation, is to a level not different than that seen in control kidneys at 0 hour, and when measured by direct kidney histology, is profound, (3) PrC-210 provides 100% protection to naked DNA against an ·OH insult, whereas all other existing antioxidants in UW solution, or proposed antioxidants for new preservation solutions, were without effect, and (4) complete protection of rat kidney mitochondria against lipid peroxidation and mitochondrial DNA fragmentation was conferred by 2 mmol/L PrC-210, a concentration known to be achieved and tolerated in the plasma of PrC-210-protected animals.

The kidney cell death associated with extended CI storage, alone, is substantial. At 30-hour storage, there was a 51% increase in kidney-activated caspase activity (Figure 1A). This is sizable when compared with the 67% increase in activated caspase we observed in an earlier study 24 hours after a 30-minute ischemia (ligation) and warm reperfusion (ligation release) in the mouse kidney model.^24^ Transplanted solid organs receive both of these insults, together. An agent, PrC-210, that has been shown to reduce both of these insults, essentially to background, would clearly be expected to confer a substantial improvement in the solid organ transplant process.

Significant suppression of both CI and warm-reperfusion would be expected to (1) reduce both the incidence and substantial costs of managing a patient with delayed graft function, for example, dialysis, hospital days, surgery costs, organ failure, and removal and (2) increase the pool size of available organs to transplant.

Normal mitochondrial oxidative function, and more importantly, its aberration under hypoxic CI storage and hyperoxic reperfusion conditions,^31^ is a significant determinant of how oxygen and its free-radical forms cause injury to cells. So it is significant that (1) PrC-210 conferred complete suppression of both lipid peroxidation and mitochondrial DNA fragmentation and (2) it did so at a concentration (2 mmol/L) that has been readily achieved in the plasma of both mice and rats that were given either intraperitoneal or oral systemic 0.5 maximum tolerated dose doses of PrC-210 that were tolerated with no detectable toxicities.

Although the addition of 30 mmol/L PrC-210 to D-PBS conferred a significant reduction in caspase activation over the 30 hours of CI storage, D-PBS as a preservative is clearly inferior to UW solution as an organ preservation solution.

**FIGURE 7.**
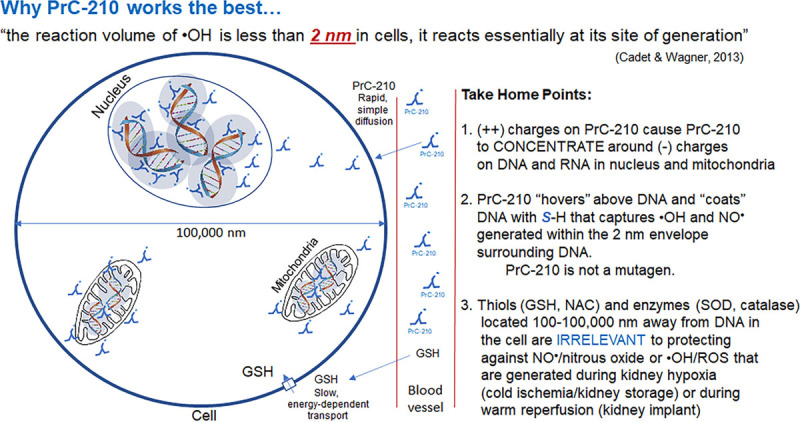
Schematic of PrC-210 mechanism of action, which involves (1) rapid and simple diffusing across membranes for the MW: 146 molecule, (2) concentrating in cells around (−) charged DNA and RNA in nuclei and mitochondria, and (3) extremely efficient ROS-scavenging with its thiol nucleophile. NO·, nitric oxide; ROS, reactive oxygen species.

An important advantage of PrC-210, which is not an antioxidant, is its immediate action as an ROS scavenger, summarized in Figure 7. Its (1) small size, (2) simple transmembrane diffusion, and (3) (+) charges all serve to place it around (−) charged nucleic acids and proteins in nuclei and mitochondria. These PrC-210 characteristics explain why it is effective in capturing the ·OH produced in cells, especially in light of the reported reaction radius of 2 nm for ·OH.^32^

The active PrC-210 thiol form half-life at pH 7.2 is 3.5 hours.^23^ Perfusion at 0 hour with 30 mmol/L PrC-210 in UW solution (Figure 5A) confers complete kidney protection over 30 hours of 4°C storage. This indicates that ≈4 half-lives at pH ≈7.2 (30 → 1.9 mmol/L PrC-210 thiol) still provides a PrC-210 thiol concentration >1.5 to 2 mmol/L PrC-210 thiol seen in the plasma of mice that received 100% protection against an otherwise 100% lethal dose of whole-body irradiation.

Finally, the ability of PrC-210 to completely suppress both CI kidney damage over 30 hours (Figure 5A), and warm-reperfusion kidney injury,^24^ would allow PrC-210 to be used in a variety of transplant and surgical settings to reduce IR injury. PrC-210 could be (1) added to preservation solutions and perfused through an organ, (2) injected directly into the organ before implant, or (3) be given intravenously to both organ donor and recipient patients. Importantly, the nature of PrC-210 as a direct-acting, highly effective ROS scavenger would also allow it to be used in any environment in which blood flow is stopped and restarted, such as coronary bypass surgeries, neurological procedures following stroke, and during aorta aneurysm repairs.
